# Evaluation of Antioxidant Activities from a Sustainable Source of Okara Protein Hydrolysate Using Enzymatic Reaction

**DOI:** 10.3390/molecules28134974

**Published:** 2023-06-24

**Authors:** Andriati Ningrum, Dian Wahyu Wardani, Nurul Vanidia, Manikharda, Achmat Sarifudin, Rima Kumalasari, Riyanti Ekafitri, Dita Kristanti, Woro Setiaboma, Heli Siti Helimatul Munawaroh

**Affiliations:** 1 Department of Food and Agricultural Product Technology, Faculty of Agricultural Technology, Universitas Gadjah Mada, Flora Street No. 1, Bulaksumur, Yogyakarta 55281, Indonesia; 2 Research Centre for Appropriate Technology, National Research and Innovation Agency, KS. Tubun Street No. 5, Subang 41213, Indonesia; 3 Research Center for Food Technology and Processing, National Research and Innovation Agency, Jogja-Wonosari Street km 31, 5 Playen, Gunungkidul, Yogyakarta 55861, Indonesia; 4 Study Program of Chemistry, Department of Chemistry Education, Faculty of Mathematics and Science Education, Universitas Pendidikan Indonesia, Bandung 40154, Indonesia

**Keywords:** okara, peptide, antioxidant, in vitro, valorization, papain

## Abstract

Okara is a solid byproduct created during the processing of soy milk. The production of protein hydrolysates utilizing enzymatic tests such as papain can result in the production of bioactive peptides (BPs), which are amino acid sequences that can also be produced from the okara protein by hydrolysis. The objective of this study was to investigate the antioxidant activities of okara hydrolysates using papain, based on the in silico and in vitro assays using the papain enzyme. We found that using the in silico assessment, the antioxidant peptides can be found from the precursor (glycinin and conglycinin) in okara. When used as a protease, papain provides the maximum degree of hydrolysis for antioxidative peptides. The highest-peptide-rank peptide sequence was predicted using peptide ranks such as proline–histidine–phenylalanine (PHF), alanine–aspartic acid–phenylalanine (ADF), tyrosine–tyrosine–leucine (YYL), proline–histidine–histidine (PHH), isoleucine–arginine (IR), and serine–valine–leucine (SVL). Molecular docking studies revealed that all peptides generated from the parent protein impeded substrate access to the active site of xanthine oxidase (XO). They have antioxidative properties and are employed in the in silico approach to the XO enzyme. We also use papain to evaluate the antioxidant activity by using in vitro tests for protein hydrolysate following proteolysis. The antioxidant properties of okara protein hydrolysates have been shown in vitro, utilizing DPPH and FRAP experiments. This study suggests that okara hydrolysates generated by papain can be employed as natural antioxidants in food and for further applications, such as active ingredients for antioxidants in packaging.

## 1. Introduction

Okara is the leftover soybean residue from the production of soymilk or soybean curd. Because of its high moisture content (70–80 percent), it is easily spoiled and frequently thrown. Nevertheless, okara still maintains numerous nutrients (on a dry weight basis, roughly 50 percent carbohydrates, 20–30 percent proteins, and 10–20 percent lipids, as well as minerals and phytochemicals), making it a viable substrate for biovalorisation [1]. Okara’s high protein content makes it an excellent candidate for development as a source for functional components such as bioactive peptides. In the food business, bioactive peptides in the form of hydrolysates are utilized as food additives because they have good functional qualities in food items, such as binding water or oil, emulsifying, gelling, or foaming agents. These functional qualities are features other than the nutritional value that impact food sensory reception, particularly the physicochemical properties of proteins [2]. On the other hand for other applications, the protein hydrolysates also have potential as active antioxidant agents in packaging applications [3]. The importance of several bioactive peptides is also very important for human health. Peptides from several sources have been reported to favorably affect immune (antimicrobial, immunomodulatory, and anti-cancer), cardiovascular (antithrombotic, antidiabetic, antihypertensive, hypolipidemic, anti-inflammatory), nervous (antinociceptive, relaxing, anti-amnesic), and gastrointestinal (anti-obesity) health [4,5]. 

In silico approaches using BIOPEP and PeptideRanker were employed to predict and analyze the theoretical antioxidative peptides from the parent protein. Research on the in silico approach is also very important to understand the protein–ligand interactions [6,7,8]. The frequency of bioactivity of the peptide fragments generated by specified enzymes and the theoretical degree of hydrolysis were used to calculate the quantitative value of bioactive peptides derived from collagen (DHt). To assess the hydrolysate’s additional bioactivity, numerous dietary sources were hydrolyzed in silico to generate bioactive peptides [9,10,11].

Despite the widespread usage of the in silico approach, experimental hydrolysis is still necessary to prove the possibility of bioactivity based on in silico data. In vitro enzymatic hydrolysis can result in three major changes in the protein: a reduction in molecular weight, increased availability of hydrophobic groups, and ionic group release. These modifications have an impact on the antioxidant capabilities of peptides [12]. The efficient integration of in silico and in vitro protein-derived peptides was validated by the related antioxidant bioactivity data [12]. Since most of the lifestyle-related disease pathologies are associated with oxidative stress, antioxidant peptides that can stimulate a better physiological stress response, while exerting other preferred bioactivities, hold promise for health improvement.

The papain enzyme was used to make the okara hydrolysate in this investigation. The papain enzyme was chosen because it is an endo/exoprotease enzyme that can catalyze the hydrolysis process at specific linkages. Because of the potential for papaya production in tropical nations, particularly Indonesia, the papain enzyme is synthesized from the papaya isolate and may be used for generating bioactive peptides [13]. In this work, we will assess the hydrolysate of okara protein in silico and in vitro using the papain enzyme. The in silico antioxidant activity of several chosen key bioactive peptides discovered in okara hydrolysates was examined using molecular docking on oxidative-stress-related molecular targets, including xanthine oxidase (XO) [10,11,12]. In vitro, antioxidant capacity will be assessed utilizing the DPPH and FRAP techniques. Thus, the study aims to explore the bioactivity of okara hydrolysate for antioxidant activity through in silico and in vitro integration approaches.

## 2. Results and Discussion

### 2.1. The Selection of Optimum Protease Based on In Silico Analysis

**Figure 1:**
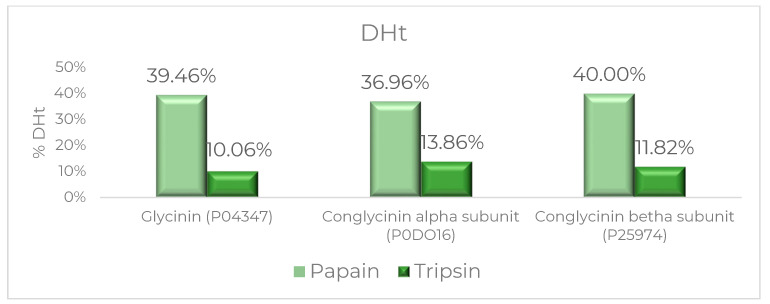
Prediction of the DHt of okara protein precursors using papain and trypsin in an in silico approach.

By using an in silico approach (BIOPEP), we tried to investigate and find the potential bioactivity properties of peptides (Table 1). Several amino acid compositions of the protein precursor in okara, e.g., glycinin and conglycinin, can be seen in Table 2. The sequences of glycine and conglycinin chains were obtained from NCBI (https://www.ncbi.nlm.nih.gov/ (accessed on 1 May 2023)) and submitted to BIOPEP for in silico proteolysis. After we found that the bioactive peptide from glycinin and conglycinin from okara soybean residue has antioxidant activity, we tried to evaluate several functions of protease, e.g., papain and trypsin, to give an optimum degree of hydrolysis. Based on Figure 1, papain can be selected as one of the optimum enzymes to produce antioxidant peptides with the highest activity. Papain was the proteases enzyme that was selected for further analysis in vitro. Previous research also showed that papain is a potential protease for generating bioactive peptides from the parent protein of the food matrix [12,13,14].

### 2.2. Peptide Ranking

The bioactivity of okara-soybean-derived antioxidant peptides was calculated using the peptide ranker (http://distilldeep.ucd.ie/PeptideRanker/ (accessed on 1st May 2023)). The bioactivity score ranged from 0 to 1, with the highest score (1) indicating the most active peptides, and the lowest score (0) indicating the least active peptides. Table 1 displays several bioactive peptides with higher-peptide-rank okara soybean residues. With a peptide rank score of 0.94, the proline–histidine–phenylalanine (PHF) sequence had the highest peptide rank score among the antioxidant peptides of the okara soybean residues. All peptide sequences with antioxidant activity are non-toxic, and some of the taste is hardly detectable (Table 3).

### 2.3. Physiochemical Properties of Peptides

Peptide antioxidant activity is influenced by structural properties such as molecular mass, amino acid content, and the peptide sequence. Solubility, net charge, and the iso-electric point (Table 4) are important physical attributes to consider when creating new treatments, because they impact the dispersion in the human body and the capacity to target certain bacteria, cells, proteins, or viruses. As a consequence, the peptide calculator on the INNOVAGEN website estimated additional physical chemistry parameters of the proposed peptides (PEPCALC) [15], and the outcome is shown in Table 2. Peptides ADF and IR were shown to be soluble in water.

### 2.4. In Silico Peptide Docking with an Antioxidant Enzyme (Binding Affinity and Interaction)

**Figure 2:**
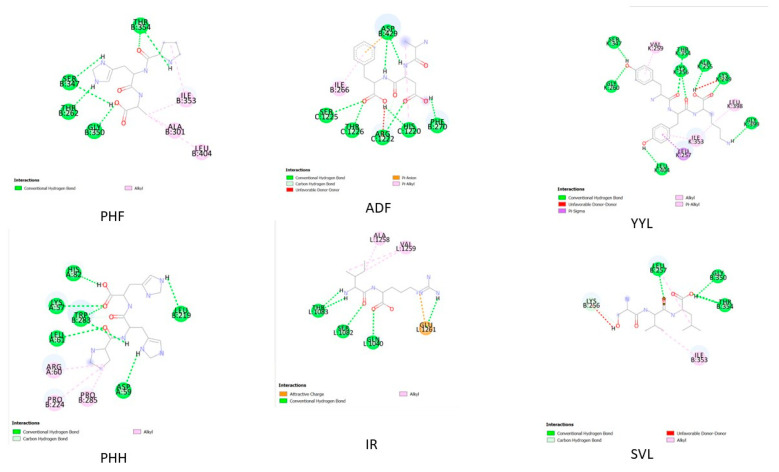
Two-dimensional interaction of the ligands and XO.

**Figure 3:**
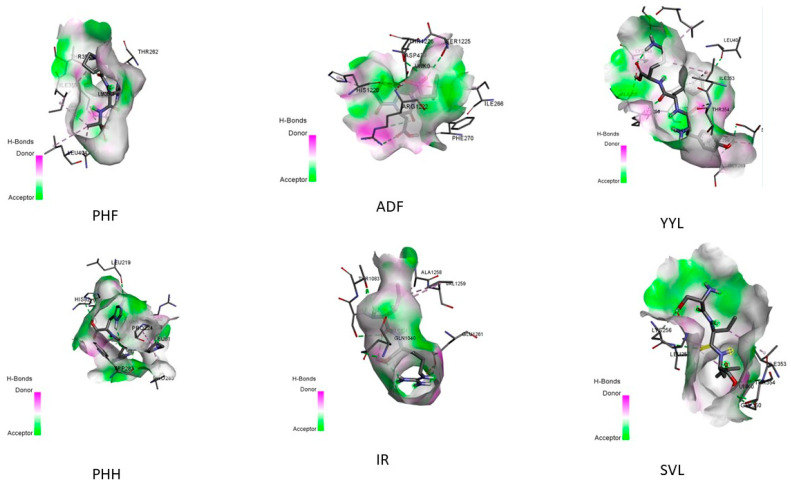
Three-dimensional interaction of the ligands and XO.

Molecular docking is a method for modeling receptor–small-molecule interactions in silico [12,16]. Several molecular docking approaches were employed to identify XO inhibitors with antioxidant activity, demonstrating the efficacy of this strategy [17]. As a result, molecular docking may be used to find antioxidants and uncover the underlying molecular processes. All peptides derived from glycinin and conglycinin degraded with papain spontaneously docked onto XO, based on the binding affinity assessed by AutoDock Vina (Table 5). Peptides produced from glycinin and conglycinin have a high affinity for XO, and the molecular interactions between peptides and XO include hydrogen bonds, hydrophobic interactions, and electrostatic interactions (Figure 2). The existence of hydrogen bonds and hydrophobic interactions suggests the peptide–protein complex stability. One of the most important molecular interactions is hydrogen bonding, which helps maintain the three-dimensional structure of proteins and nucleic acids [18]. Furthermore, all okara protein peptides, such as glycinin and conglycinin, interact with key XO residues (Figure 3). Peptides are far more flexible than small molecules and proteins, and do not have a stable shape before interacting with a receptor [19].

**Figure 4:**
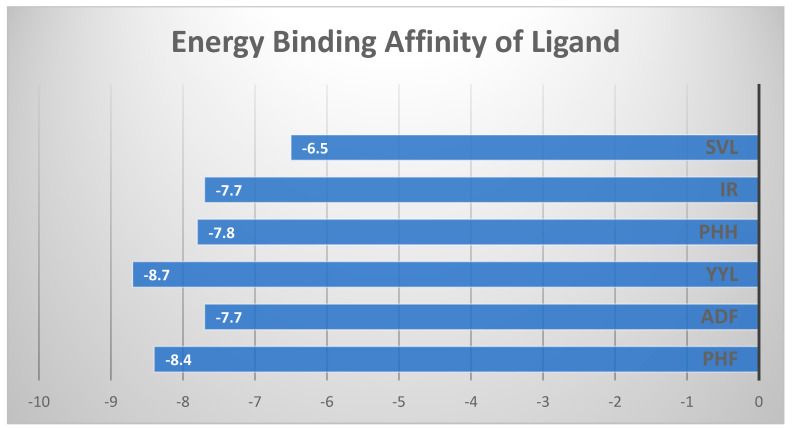
Energy binding affinity of the ligands (peptides) against XO.

Figure 2 and Figure 3 depict the interaction between the six peptides with XO, with binding energies of –6.5 and −8.7 kcal/mol (detailed in Figure 4), respectively, indicating a strong binding relationship. Generally, the lower the binding energy for the same docking model, the more stable the complex. In this study, we employed energy binding affinity to attach okara peptides to XO, to efficiently analyze peptide flexibility. To reflect the effect of molecular docking, more negative energy was employed (Figure 4). The (−) energy value shows the XO–peptide binding affinity, with a higher value suggesting a more attractive combination [20].

Xanthine oxidase is an enzyme that catalyzes the oxidation of hypoxanthine to xanthine, which is then oxidized to uric acid by the enzyme. Because of its capacity to generate reactive oxygen species such as superoxide and hydrogen peroxide, the enzyme is classed as an oxidoreductase. The inhibition of the enzyme’s activity by xanthine oxidase inhibitors is predicted to cure hyperuricemia, reduce oxidative stress, and prevent pathological inflammatory responses [21]. As a consequence, molecular docking was used to see if the different peptides were compatible. The energy of the peptides, as shown in Figure 4, demonstrated that the peptides may spontaneously attach to the XO [22].

In theory, inhibitory peptides can inactivate enzymes by occupying active sites or obstructing the entry of the active site cavities. The XO docking postures of the peptides are shown in Figure 4, and both of them blocked the entry to the XO active cavity, demonstrating that they are potent antioxidants. Among the interactions between XO and peptides were hydrogen bonds. As a result, the in silico antioxidant activity of okara-derived peptides may be in part due to the XO inhibitory effects.

Molecular docking is an in silico approach for simulating receptor–small-molecule interactions [23]. To choose XO inhibitors with antioxidant activity, many molecular docking approaches were used, proving the usefulness of this strategy. As a result, molecular docking is an effective tool for identifying antioxidants and elucidating the underlying molecular processes. Based on the energy binding affinity determined by Pyrix, all peptides generated from glycinin and conglycinin spontaneously docked onto XO. Peptides generated from glycinin and conglycinin have a binding affinity for XO, and the molecular interactions between the peptides and XO include hydrogen bonds, covalent bonds, and numerous protein–ligand interactions (Figure 4). The presence of hydrogen bond interactions shows the stability of the complexes formed between the peptide and protein. The hydrogen bond, which stabilizes the three-dimensional structure of proteins and nucleic acids, is one of the most important interactions between molecules. 

The forces created between the peptides and XO (PDB: 3NVY), as well as the inter-actions and matching bonds, are depicted in Figure 2 and Figure 3, and Table 5. The peptides interacted with the protein, and for detailed interaction of peptides can be seen in Table 5. PHF had hydrogen connections established with THR354, SER347, THR262, and GLY350. PHF also had hydrophobic interactions established with ALA301, ILE353, and LEU404. ADF had hydrogen connections established with PHE270, HIS1220, ARG1222, THR1226, and SER1225. ADF also also had hydrophobic interactions established with ILE266 and electrostatic interactions with ASP 429. YYL had hydrogen connections established with HIS82, LYS57, TRP283, LEU61, ASP59, and LEU219. YYL also had hydrophobic interactions established with ARG60, PRO224, and PRO285. PHH had hydrogen connections established with HIS82, LYS57, TRP283, LEU61, ASP59, and LEU219. On the other hand, PPH had hydrophobic interactions established with ARG60, PRO224, and PRO285. IR had hydrogen connections established with THR1083, THR1082, and GLN1040. On the other hand, IR had hydrophobic connections established with ALA1258 and VAL1259, and electrostatic connections with GLU1261. SVL had hydrogen connections established with LEU257, GLY350, and THR354. SVL also had hydrophobic connections established with ILE353 and LYS256. Peptides are far more flexible than tiny molecules and proteins, and do not have a fixed shape before interacting with a receptor [24]. The outcome of molecular docking was reported as (−) energy binding activity (Figure 4). The (−) energy value reflects the bonding affinity between XO and peptides, with a greater value indicating a more suitable combination [25].

### 2.5. Proximate Analysis

The water, crude protein, lipid, carbohydrate, fiber, and ash content of the okara biomass were determined using a proximate analysis approach. Wet okara has a water content of 82.8 percent, a protein content of 6.34 percent, a lipid content of 0.86 percent, and an ash content of 0.65 percent. Dried okara includes 3.47 percent water, 36.10 percent protein, 13.60 percent fat, and 3.25 percent ash after drying in a cabinet dryer. 

### 2.6. Yield

**Figure 5:**
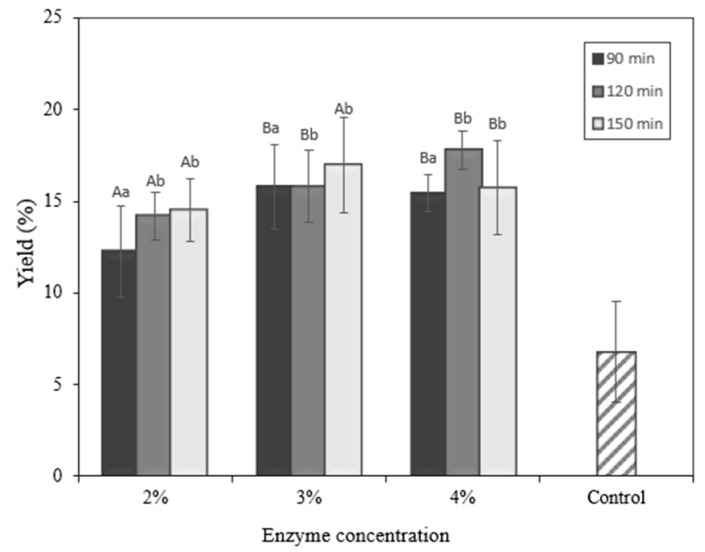
Yield of okara hydrolysate. ^A,B^ Different capital letters indicate a statistical difference between the enzyme concentrations (*p* < 0.05). ^a,b^ Different non-capital letters indicate a statistical difference between the times of hydrolysis (*p* < 0.05). Values represent the mean ± standard error.

**Figure 6:**
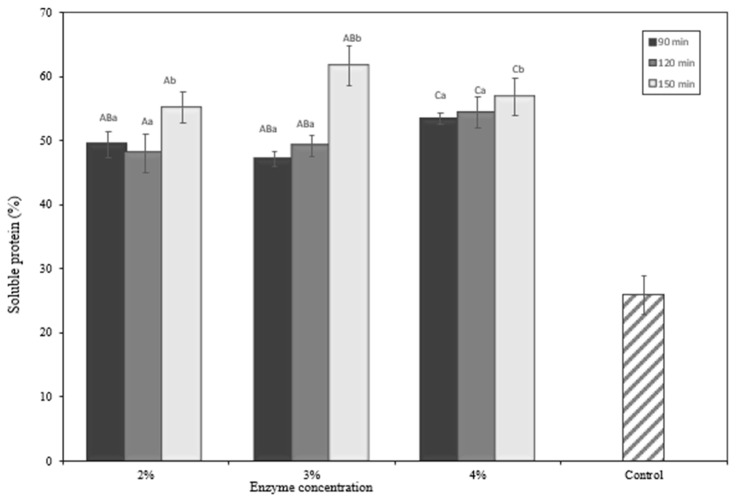
Total soluble protein of okara hydrolysate. ^A–C^ Different capital letters indicate a statistical difference between the enzyme concentrations (*p* < 0.05). ^a,b^ Different non-capital letters indicate a statistical difference between the times of hydrolysis (*p* < 0.05). Values represent the mean ± standard error.

To evaluate its potential for larger-scale production, the yield and productivity of the protein hydrolysate from okara in the form of freeze-dried solids were estimated. Figure 5 depicts the yield of the protein hydrolysate. The total soluble protein is strongly influenced by the enzyme concentration and hydrolysis time (*p* < 0.05). A previous study developed solid protein hydrolysates from *Hermetia illucens*, with yields varying depending on the hydrolysis setting [26]. Protein hydrolysate yield varies based on the hydrolysis parameters used to increase hydrolysis yield. Okara’s total soluble protein may also be seen in protein hydrolysate yield, varied based on the hydrolysis parameters used to increase the hydrolysis yield. Okara’s total soluble protein may also be seen in Figure 6. Hydrolysis of okara was performed only up to 150 min in this study, considering economic efficiency. Overall, the yield increased as the hydrolysis time was prolonged for okara hydrolysates prepared by papain (Figure 5). Comparing the yield among different types of enzyme concentrations and hydrolysis times, the optimum was reached at 4% enzyme concentration and 120 min.

### 2.7. Degree of Hydrolysis

**Figure 7:**
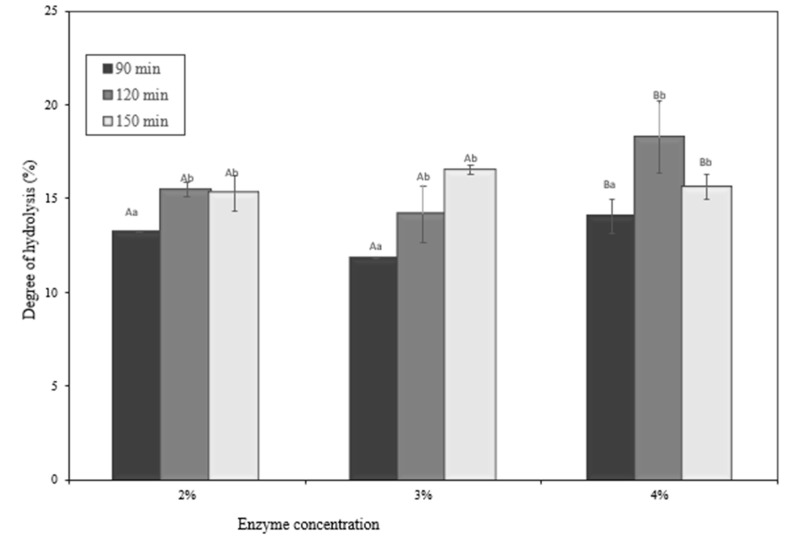
Degree of hydrolysis of okara hydrolysate. ^A,B^ Different capital letters indicate a statistical difference between the enzyme concentrations (*p* < 0.05). ^a,b^ Different non-capital letters indicate a statistical difference between the times of hydrolysis (*p* < 0.05). Values represent the mean ± standard error.

The determination of the degree of hydrolysis is explained in Figure 7. According to Figure 7, the quantity of papain and the duration of the time affect the degree of hydrolysis (*p* < 0.05). The okara protein hydrolysate was effectively synthesized using a range of manufacturing techniques, with the DH values varying (Figure 7). This discovery demonstrates that the DH value is controlled by a variety of parameters, including the enzyme concentration and hydrolysis time [26,27]. Based on Figure 7 representing the DH, the concentration of an enzyme can influence the degree of hydrolysis. The value of the degree of hydrolysis (DH) indicates a decrease in the estimate of the protein particle due to the continuous assimilation of protein enzymatic breakdown causing the breakdown of the intact protein structure into littler peptides and amino acids as a result of peptide bond cleavage [28]. Papain, as a protease, breaks down amide groups within proteins by breaking peptide bonds. The catalysis process takes place in the cysteine group (Cys-25), which is extremely reactive and binds to the substrate on the active side of papain, resulting in a tetrahedral covalent link between the substrate and the enzyme. The histidine group (His-159) connects to the nitrogen in the substrate, causing the amine group to disperse and be replaced by water molecules, which eventually hydrolyze the intermediate product and restore the enzyme to its original shape and activity. The second reaction, based on the mechanism of enzyme binding to the substrate, is a deacylation reaction, which is characterized by the hydrolysis of the enzyme–substrate complex bonds into products and enzymes [29]. In assessing the functional characteristics of the hydrolyzed proteins, the degree of hydrolysis is an important signal. The enzyme concentration and process duration had a substantial impact on the degree of hydrolysis. As the degree of hydrolysis increased and the peptide bands were broken, the chain length of the peptides got shorter. As a result, the number of free amino acids rose. This discovery was consistent with the findings of an earlier study [30]. The DH (degree of hydrolysis) is defined as the percentage of cleaved peptide bonds after hydrolysis, and it is a critical factor that contributes to the composition and functional properties of the peptides. Comparing the DH between the different concentrations of enzyme and hydrolysis times demonstrated the highest DH value at a 4% enzyme concentration and 120 min. The difference in efficiency may be caused by their different specificities of cleaving peptides due to the enzyme concentration and length of hydrolysis time. The higher the enzyme concentration and the length of hydrolysis time, the higher the value of the degree of hydrolysis, which is aligned with previous research [31].

### 2.8. In Vitro Evaluation of Antioxidant Activity in Okara Hydrolysates

**Figure 8:**
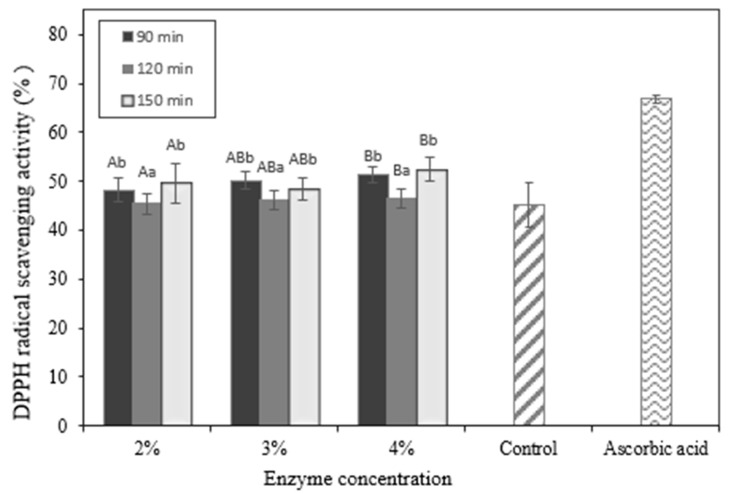
DPPH radical scavenging of okara hydrolysate. ^A,B^ Different capital letters indicate a statistical difference between the enzyme concentrations (*p* < 0.05). ^a,b^ Different non-capital letters indicate a statistical difference between the times of hydrolysis (*p* < 0.05). Values represent the mean ± standard error.

**Figure 9:**
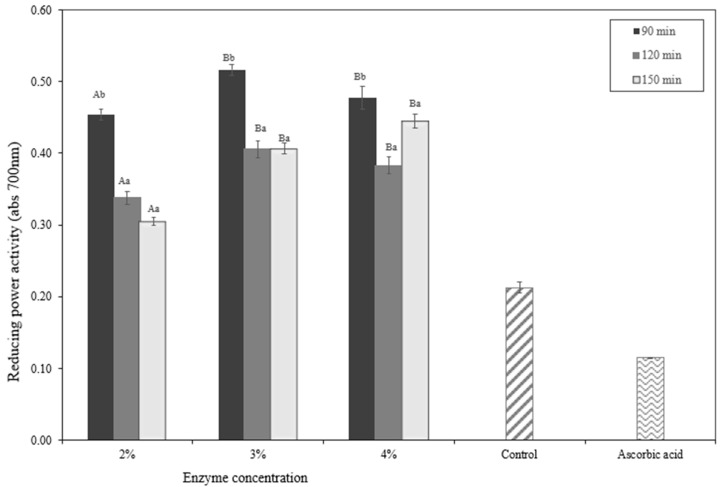
FRAP analysis of okara hydrolysate. ^A,B^ Different capital letters indicate a statistical difference between the enzyme concentrations (*p* < 0.05). ^a,b^ Different non-capital letters indicate a statistical difference between the times of hydrolysis (*p* < 0.05). Values represent the mean ± standard error.

The in vitro evaluation of antioxidant activity measurements of okara hydrolysates may be observed in Figure 8 and Figure 9. The quantity of the enzyme and the period of hydrolysis both impact antioxidant activity. The stronger the antioxidant, the greater the amount of enzyme and the length of time. According to the DPPH free-radical scavenging study and FRAP, both the okara protein hydrolysate and the control sample exhibited antioxidant activity, the hydrolysate demonstrated significantly higher levels of activity.

A protein hydrolysate’s antioxidant activity is the consequence of several synergistic mechanisms, including free-radical deterrence or suppression, prevention of lipid peroxidation processes, metal ionization, countering oxidative reactions by oxygen-containing chemicals, and electron transfer activities [26]. Because DPPH radicals are purple, samples with antioxidants will change color to a lighter tone [32]. Based on Figure 8 that shows antioxidant activities according to DPPH radical scavenging activities, the higher enzyme concentration and longer the hydrolysis time tend to produce a higher antioxidant activity. This finding is also in line with previous studies concluding that the raw material of okara, which is soybean, also has antioxidant activities [33].

The DPPH free radical inhibition activity of okara protein hydrolysate is also impacted on by numerous parameters, including the amino acid content. Aromatic amino acids, such as phenylalanine and tyrosine, can stabilize DPPH free radicals by acting as proton donors for electron-deficient free radicals. The proton donor mechanism causes the free-radical reaction to propagate and end. Furthermore, hydrophobic amino acids perform a crucial part in the process of repelling free radicals [12]. This is due to the capacity of hydrophobic amino acids to promote free-radical access to related amino acids, hence lowering free-radical access in attacking chemicals and targeted cells [12]. Thus, okara protein hydrolysate has the potential to be a bioactive hydrolysate with antioxidant activity that can block free radicals such as DPPH for its antioxidant activities.

The reduction capacity of the peptide was computed based on the electron donation by the residue amino acids, particularly the sulfhydryl groups of Cys, and was specifically tested by measuring the reduced state of the Fe(III)/Fe(II) redox pair [34]. Based on this study, the concentration of the papain enzyme and the length of time can influence the antioxidant activity of okara hydrolysate (*p* < 0.05). This study contributes to developing the protein hydrolysate for a broad range of applications. 

Samples were selected for peptide sequencing by LCMS (Table 6). Tyrosine (Y), tryptophan (W), methionine (M), lysine (K), and cysteine are some amino acids that exhibit antioxidant properties (C). Through resonance structures, aromatic residues in amino acids can contribute protons to electron-deficient radicals while keeping their stabilities. This characteristic boosts the amino acid residues’ radical scavenging capability. Aromatic amino acids were found in both enzymatic hydrolysates and certain peptides with aromatic residues at the carboxyl terminus. We also confirmed that several peptide sequences analyzed using LC-MS also aligned with the in silico assays. Thus, this method confirming those peptides contributes to the knowledge on the functionality for the antioxidative effect, especially in promoting okara for functional ingredients. As previously investigated in our experiments, here we show that the complementing in vitro screening with in silico testing can not only increase the number of bioactivity testing carried out, but also provide an opportunity to narrow down bioactive peptide prospects with the use of predictive platforms such as for physicochemical characteristics, toxicity, and sensory evaluation of the predicted peptides [4]. In the era of big data, the use of the bioinformatics-based approach helps save a significant amount of resources while casting a bigger net for prospective bioactive peptides from okara-derived protein precursors. Using in silico platforms makes the bioactive peptides much faster and cheaper for further functional food applications or nutraceutical drug shortlists, making the functionality discovery approach of this bioactive peptide more efficient [4]. A comprehensive study on the integrated in silico and in vitro approach to the antioxidant activity produced, especially for the antioxidant activity of hydrolysates and peptide fractions from okara as the food industry by-product, contributes to the development of the protein hydrolysate from okara in food and health applications. Alongside that, it can also be incorporated into the development of sustainable packaging as an active compound. Several peptides are already used as active compounds incorporated into food packaging [35].

## 3. Materials and Methods

### 3.1. Material

Okara, as the research material, was purchased from the Soymilk Small Medium Enterprise in Yogyakarta, Indonesia. Okara was kept in a freezer at −40 °C using an airtight jar. 

### 3.2. Enzyme and Chemicals

Papain (Himedia, Thane, India), trichloroacetic acid (TCA), 2-2diphenyl-1-picrylhydrazyl (DPPH), and sodium hydroxide (NaOH) were used (Merck, Darmstadt, Germany, Hydrochloric Acid) and all chemicals were of analytical grade. 

### 3.3. In Silico Analysis

#### 3.3.1. The Selection of Optimum Protease Based on In Silico Analysis

The optimal protease for generating antioxidant peptides from glycinin and conglycinin as parent proteins in okara was chosen using an in silico technique. Proteases with a high antioxidant frequency and degree of hydrolysis were utilized as enzymes during in vitro proteolysis. The NCBI sequences of glycinin and conglycinin were utilized for the parent protein (https://www.ncbi.nlm.nih.gov/ (accessed on 1 may 2023)). Based on information about the sequences from the NCBI database, the sequences were analyzed using the BIOPEP database (https://biochemia.uwm.edu.pl/en/biopep-uwm-2/ (accessed on 1 May 2023)). Proteolysis parameters determined the theoretical degree of hydrolysis (DHt).

#### 3.3.2. Peptide Ranking

The peptide ranker (http://bioware.ucd.ie/compass/biowareweb/Serverpages/peptidaranker.p (accessed on 1 May 2023)) was used to determine the bioactivity of the chosen glycinin and conglycinine antioxidant peptides. The bioactivity score ranged from 0 to 1, with the highest score (1) indicating the most active peptides and the lowest score (0) indicating the least active peptides.

#### 3.3.3. Toxicity and Sensory Prediction

The BIOPEP database was used to research the toxicity and sensory prediction. Other peptide sequence characterization was estimated using a peptide calculator (https://pepcalc.com/peptide-solubility-calculator.php (accessed on 1 May 2023)).

#### 3.3.4. Molecular Docking against Peptides

The software AutoDock Vina-4.2.6 was used to conduct a molecular docking investigation. The crystal structure of bovine xanthine oxidase (PDB ID: 3NVY) was retrieved from the RCSB Protein Data Bank (https://www.rcsb.org/ (accessed on 1 may 2023)), and the peptides derived were retrieved from PubChem. Both the protein and peptides were prepared using PyRix. The activity was generated through the global docking method. The visualization of docking data was shown using LigPlus (2D), PyMol (3D), and Biovia discovery (3D).

### 3.4. Preparation of Okara Hydrolysates Using Enzymatic Hydrolysis

Fresh okara was dried in a cabinet at 50 °C for 48 h. Okara flour (10 g) was mixed with 200 mL of water. The hydrolysis temperature was carried out at 40 °C. Before initiating hydrolysis, the mixture was preincubated at 40 °C for 20 min for preconditioning the temperature. After preconditioning the temperature, the enzyme papain was added at 2%, 3%, and 4% (*w*/*v*), and the incubation time at 90, 120, and 150 min. To deactivate the enzyme, the mixture was heated to 85 °C for 10 min. As the control experiment, hydrolysis without enzymes and incubation was performed. The mixture was centrifuged at 4000 rpm at 4 °C for 20 min, and the supernatant was collected and freeze-dried (Labconco, Kansas City, MO, USA) at −50 °C under vacuum conditions.

### 3.5. Proximate Analysis

Proximate analysis was determined via AOAC, 1960. The moisture content was determined using the oven technique at 105 °C until a stable weight was obtained. The crude protein was tested using the Kjeldahl technique (N 6.25) (Kjeltec, Suzhou, China). The Soxhlet technique was used to determine the fat content. At 600 °C, the total ash content was measured using a muffle furnace (Advantec, Tokyo, Japan). All tests were carried out in triplicate [36].

### 3.6. Yield

The yield of okara hydrolysates was measured using the following equation:

$$\mathrm{Yield}\;\left(\%\right)=\frac{\mathrm{Weight}\;\mathrm{of}\;\mathrm{okara}\;\mathrm{hydrolysate}\;\left(g\right)}{\mathrm{Weight}\;\mathrm{of}\;\mathrm{okara}\;\mathrm{isolates}\;\left(g\right)}\times 100\%$$

### 3.7. Degree of Hydrolysis

The degree of hydrolysis was determined by following the procedure described in [14]. Bovine serum albumin with a concentration of 0–0.3 mg/mL was used as a standard. In the dark, 1 mL of the material was dissolved in water and mixed with reagent D. After 15 min, each aliquot was homogenized and incubated for 45 min with 3 mL of reagent E. The sample was then measured at a wavelength of 750 nm (Spectrometer Genesys 840-208100 UV, Thermo Scientific, Waltham, MA, USA).

TCA 20% was added to a 0.25 mg/mL sample in a 1:1 ratio, and homogenized using a vortex. Following homogenization, the sample was incubated at 4 °C for 30 min, before being centrifuged at 3000 rpm for 10 min in a Heraeus Megafuge 8R. The Lowry technique was used to collect and analyze the supernatant and determine the sample’s soluble protein [15] using the following equation:

DH calculated formula = TCA soluble protein/total soluble protein × 100

### 3.8. Antioxidant Assays

#### 3.8.1. DPPH Radical Scavenging Activity

Radical DPPH was determined through the inhibition of DPPH using a previously described method [12]. Of the sample, 1 mL was added to 1 mL of DPPH stock solution and shaken for 30 s. The mixture was incubated in a dark room for 1 h. After that, the mixture was measured at 517 nm (UV–Vis G10S Thermoscientific, Waltham, MA, USA). The scavenging of DPPH radicals was measured according to the following equation:

Radical scavenging activity (%) = (A blank − A sample)/A blank × 100

where A blank is the absorbance of the solution without the sample, and A sample is the absorbance of the solutions with the sample [37].

#### 3.8.2. Antioxidant Analysis (FRAP Method)

The ability to measure antioxidant activity based on the FRAP method refers to a previously described method [12]. A total of 1 mL of the dissolved protein hydrolysate sample was mixed with 1 mL of 0.2 M phosphate buffer solution (pH 6.6), 1 mL of potassium ferricyanide, and 1 mL of 1% K_3_Fe(CN)_6_. The mixture was incubated at 50 °C for 20 min, then 1 mL of 10% TCA solution was added to stop the reaction, and then centrifuged at 3000 rpm for 10 min. Of the supernatant solution, 1 mL was taken, added with 1 mL of distilled water and 0.5 mL of 0.1% FeCl_3_. The mixture was incubated again at 50 °C for 10 min. After that, the sample was calibrated at an absorbance wavelength of 700 nm (UV–Vis G10S Thermoscientific, Waltham, MA, USA). The blank contained all the reagents, except the sample. The positive control used vitamin C for the comparison.

### 3.9. Determination of the Sequence of the Peptide

Formic acid (0.1 percent) was used to dissolve the sample. Following that, 5 µL of the sample was injected. Mobile phases A (water + 0.1% formic acid) and B (acetonitrile + 0.1% formic acid) were analyzed using the Thermo Scientific^TM^ DionexTM Ultimate 3000 RSLC nano UHPLC, paired with the Thermo Scientific^TM^ Q Exactive^TM^ High-Resolution Mass Spectrometer. The acquired data were processed using Full MS 70,000 FWHM Resolution. Thermo Scientific^TM^ Proteome Discoverer 2.2 Software was used to identify proteins, which were then compared to the Master Protein database.

### 3.10. Statistical Analysis

Data were presented as the mean ± standard error (*n* = 3). A two-way ANOVA was performed using SPSS 25.0 for Windows (SPSS Inc., Chicago, IL, USA), and subsequent multiple comparisons of the means were carried out using Duncan’s multiple range test (DMRT) at a level of 95% confidence.

## 4. Conclusions

Okara hydrolysates showed antioxidant potential in the in silico and in vitro assessment. Based on the in silico assay, protein precursors in okara, such as glycinin and conglycinin, may generate several bioactive peptides with their antioxidative effect. The molecular docking of several peptides in the okara hydrolysates generated by the papain enzyme can successfully be performed using molecular docking to XO as the enzyme that contributes to antioxidant activity. After performing in silico assays to observe the antioxidant activity of okara hydrolysates, in vitro assays of antioxidant activity were carried out. We also found that the hydrolysate of the okara protein, with the addition of 4% enzyme treatment of papain and hydrolysis time of 120 min, had the highest yield and degree of hydrolysis. Following that, based on in vitro assays of antioxidant activity, the okara hydrolysates also had significantly higher activities compared to the control. The sequence of the okara hydrolysate was also confirmed using LC-MS analysis. The hydrolysate also has potential properties for further application in food matrices, such as natural antioxidant agents, and can be incorporated in sustainable active packaging or nutraceutical application.

## Figures and Tables

**Table 1:** In silico assay using papain-release peptides with antioxidant activity.

No.	Protein Precursor	Peptide ID	Sequence	Location	Monoisotop Mass	Chemical Mass
1	Glycinin (P04347)					
		7868	ADF	(365–367)	260.1367	260.2861
		9879	SVL	(227–229)	317.1944	317.3802
2	Beta-conglycinin alpha subunit (P0DO16)					
		8025	PHF	(453–455)	399.1901	399.4426
		8215	IR	(214–215)	287.1952	287.3576
3	Beta-conglycinin beta subunit (P25974)					
		3300	PHH	(87–89)	389.1807	389.4081
		7868	ADF	(92–94)	351.1425	351.3536
		7941	YYL	(132–134)	457.2205	457.5182
		8025	PHF	(287–289)	399.1901	399.4426
		8215	IR	(53–54)	287.1952	287.3576

**Table 2:** Amino acid profile of the protein precursor (glycinin and conglycinin) of okara using an in silico assay.

Amino Acids	Percentage		
	Glycinin(P04347)	Conglycinin Alpha Subunit (P0DO16)	Conglycinin Beta Subunit(P25974)
Alanin (Ala)	3.9	4.6	5.2
Arginin (Arg)	6.4	7.9	7.1
Asparagin (Asn)	6.4	6.8	7.5
Aspartic Acid (Asp)	4.7	4.6	4.8
Cysteine (Cys)	1.6	0.8	0.2
Glutamin (Gln)	8.7	7.9	7.5
Glutamic Acid (Glu)	8.1	13.1	8.4
Glycine (Gly)	7.9	4.3	4.6
Histidin (His)	2.9	1.3	1.8
Isoleusin (Ile)	3.3	5.1	5.9
Leusin (Leu)	7.9	8.9	10.9
Lysin (Lys)	3.7	6.0	4.8
Methionin (Met)	1.0	0.5	0.5
Phenylalanin (Phe)	3.5	5.0	6.6
Prolin (Pro)	7.4	6.6	5.0
Serin (Ser)	8.3	7.4	7.5
Threonin (Thr)	4.1	1.8	2.5
Tryptofan (Trp)	0.8	0.3	0
Tyrosin (Tyr)	2.9	2.5	2.7
Valin (Val)	6.6	4.5	6.4

**Table 3:** Physicochemical and sensory evaluation of bioactive peptides with antioxidant activities based on an in silico approach.

No.	Sequence	Mass (g/mol)	Score	Activity	Sensory Evaluation	Toxicity
1	PHF	399.44	0.94	Antioxidant	ND	Non Toxic
2	ADF	351.35	0.81	Antioxidant	ND	Non Toxic
3	YYL	457.52	0.60	Antioxidant	ND	Non Toxic
4	PHH	389.41	0.44	Antioxidant	ND	Non Toxic
5	IR	287.36	0.33	Antioxidant	ND	Non Toxic
6	SVL	317.38	0.13	Antioxidant	ND	Non Toxic

**Table 4:** Net-charge, pI, solubility, and the extinction coefficient of peptides derived from okara soybean residue.

No.	Sequence	Solubility	pI	Peptide Charge (pH 7)	Extinction Coefficient (M^−1^·cm^−1^)
1	PHF	Poor	8.26	0	0
2	ADF	Good	0.76	−1	0
3	YYL	Poor	3.2	0	2560
4	PHH	Poor	8.41	0.2	0
5	IR	Good	10.85	1	0
6	SVL	Poor	3.37	0	0

**Table 5:** The interaction types and amino acid residues of peptides with XO.

No.	Sequence	Hydrogen Bonding	Hydrophobic Interactions	Electrostatic Interaction
1	PHF	THR354, SER347, THR262, GLY350	ALA301,ILE353,LEU404	
2	ADF	PHE270, HIS1220, ARG1222, THR1226, SER1225	ILE266	ASP 429
3	YYL	GLY260, SER347, LEU404, THR354, LYS256, ALA255, LYS249, GLY399	VAL259, LEU398,ILE353,LEU257	
4	PHH	HIS82, LYS57, TRP283, LEU61, ASP59, LEU219	ARG60,PRO224,PRO285	
5	IR	THR1083, THR1082, GLN1040	ALA1258,VAL1259	GLU1261
6	SVL	LEU257, GLY350, THR354	ILE353,LYS256	

**Table 6:** Peptide sequence analysis using LC HRMS.

No	Protein Precursors	Sequence	Mw [Da]
1	Beta-conglycinin alpha subunit 1	GSEEEDEDEDEEQDERQFPFPRPPHQK	3299.4
2	Beta-conglycinin alpha subunit 1	EEDEDEQPRPIPFPRPQPR	2332.132
4	Beta-conglycinin alpha subunit 1	GEKGSEEEDEDEDEEQDERQFPFPRPPHQK	3613.55858
8	Beta-conglycinin alpha subunit 1	QFPFPRPPHQK	1378.73786
10	Beta-conglycinin alpha subunit 1	ESEESEDSELR	1309.53901
12	Beta-conglycinin alpha subunit 1	ESEESEDSELRR	1465.64012
18	Beta-conglycinin alpha subunit 1	QFPFPRPPHQKEER	1792.92416
19	Beta-conglycinin alpha subunit 1	KQEEDEDEEQQRESEESEDSELR	2853.17767
29	Glycinin G4	WQEQQDEDEDEDEDDEDEQIPSHPPR	3210.25262
33	Glycinin G4	ADFYNPK	854.40429
34	Kunitz-type trypsin inhibitor	IGENKDAMDGWFR	1554.70055
35	Kunitz-type trypsin inhibitor	IGENKDAMDGWFR	1538.70564
39	AAI domain-containing protein	IMENQSEELEEKQK	1734.82146
40	AAI domain-containing protein	IMENQSEELEEKQKK	1878.91133
41	AAI domain-containing protein	IMENQSEELEEKQKK	1862.91642
42	AAI domain-containing protein OS=Glycine max	IMENQSEELEEKQK	1750.81637
43	AAI domain-containing protein OS=Glycine max	IMENQSEELEEK	1494.66283
44	Sucrose-binding protein	EEEQQEQHEEQDENPYIFEEDKDFETR	3470.44149
45	Sucrose-binding protein	EREEEQQEQHEEQDENPYIFEEDKDFETR	3755.58519
47	Beta-conglycinin beta subunit	NFLAGEKDNVVR	1361.71719
51	Beta-conglycinin beta subunit	ESYFVDAQPQQKEEGSKGR	2183.03635
53	Beta-conglycinin beta subunit	NFLAGEKDNVVR	1361.71719
56	Beta-conglycinin beta subunit	ESYFVDAQPQQKEEGSKGR	2183.03635
59	Lipoxygenase	EIFRTDGEQALK	1406.72742
60	Lipoxygenase	SAWMTDEEFAR	1342.57323
61	Lipoxygenase	ISPIPVLK	866.57097
62	Lipoxygenase	EEELHNLR	1039.5167
63	Lipoxygenase	YREEELHNLRGDGTGER	2030.96385
64	Lipoxygenase	YREEELHNLR	1358.68114
67	Protein disulfide isomerase	EADGIVDYLKK	1250.66269
72	Glycinin G1	FEELNNDLFR	1296.62189
75	Late embryogenesis abundant protein LEA	KLEIDDDLKLR	1357.76856
77	Lipoxygenase	SAWMTDEEFAR	1342.57323
78	Lipoxygenase	ELFRTDGEQVLK	1434.75872
83	Glucose and ribitol dehydrogenase-like	GHEDRDKDDTLK	1428.671

## Data Availability

Data will be provided under the request.

## Sample Availability

The authors do not have samples of the compounds for distribution.
